# Comparison of the Effects of Regular Periods of Immobilization and Prolonged Immobilization on Hand Function Post Distal Radial Fracture

**DOI:** 10.7759/cureus.30986

**Published:** 2022-11-01

**Authors:** Mohammed Khashab, Ahmed Alem, Alhanouf Almuatiri, Fatmah Rasheed, Mai Almehmadi, Shahad Felemabn, Samah Gassass, Majed Alosaimi, Hani Sulimani, Ali Alyami

**Affiliations:** 1 Orthopedics, King Abdulaziz Medical City, Jeddah, SAU; 2 Research Office, King Abdullah International Medical Research Center, Jeddah, SAU; 3 Orthopedics, College of Medicine, King Saud bin Abdulaziz University for Health Sciences, Jeddah, SAU; 4 Orthopaedic Surgery, King Saud Bin Abdulaziz University for Health Sciences College of Medicine, Jeddah, SAU; 5 Occupational Therapy, King Saud Bin Abdulaziz University, Jeddah, SAU; 6 Health Sciences, King Saud Bin Abdulaziz University, Jeddah, SAU; 7 Orthopaedic Surgery, King Abdulaziz Medical City/Ministry of National Guard - Health Affairs, Jeddah, SAU; 8 Orthopaedic Surgery, King Abdullah International Medical Research Center, Riyadh, SAU; 9 Orthopaedic Surgery, King Saud Bin Abdulaziz University for Health Sciences, Jeddah, SAU; 10 Orthopaedics, King Saud Bin Abdulaziz University for Health Sciences College of Medicine, Jeddah, SAU; 11 Orthopaedics, King Abdulaziz Medical City/Ministry of National Guard - Health Affairs, Jeddah, SAU; 12 Surgery/Musculoskeletal Oncology, Limb Reconstructive Surgery, Sport Medicine and Arthroscopy, King Saud Bin Abdulaziz University for Health Sciences, Jeddah, SAU

**Keywords:** hand bones - immobilization - joint range of motion - wrist injuries, wrist injuries, cast immobilization, hand-wrist bones, fracture radius

## Abstract

Introduction

Distal radius fracture (DRF) is one of the most common orthopedic cases managed in the emergency room. DRF treatment is either non-operative or operative. Regardless of the treatment methodology, a period of immobilization of 4-6 weeks is required.

Purpose

The study aims to evaluate hand function for patients who sustained DRF with different immobilization periods in King Abdul-Aziz Medical City, National Guard Hospital - Jeddah (NGHA) from December 2016 until December 2019.

Materials and methods

This is a retrospective cohort study where we collected data of DRF patients managed in NGHA. Data was collected directly from NGHA medical records (December 2016-December 2019). A total of 44 patients met the inclusion criteria. Patients were divided into two groups; a group that was immobilized as per protocol (six weeks) and a group that deviated from protocol and immobilization exceeded six weeks. A data collection sheet included the patient’s demographics, history, fracture description, management method, and hand function measurements.

Results

Of the 44 participants, 24 (54%) deviated from protocol; the remaining 20 (46%) were immobilized as per protocol. The prolonged immobilization group had limitations in hand function, restriction in extension (P-value = 0.641), and a decrease in grip strength (P-value = 0.291) compared to the per-protocol group. Flexion and radial deviation were affected similarly in both groups.

Conclusion

Although the results were not significant, immobilization for more than six weeks is associated with decreased hand function, range of motion (ROM), grip strength, and higher pain scores based on occupational therapy (OT) measurements.

## Introduction

Distal radius fracture (DRF) accounts for up to 20% of all orthopedic fractures treated in the emergency department. DRF represents 8%−15% of all bony injuries in the adult population. The magnitude of the injury depends on multiple factors, including the mechanism of injury and the energy involved [1-4]. The usual fracture mechanism of injury in the elderly is falling on an outstretched hand from a standing height with the wrist in dorsiflexion. DRF also occurs in a high-energy trauma setting, which usually is more prevalent in the younger population.

Eponyms were used initially to classify and describe DRF. Colles, in 1814 described DRF as an extra-articular, dorsally displaced fracture associated with shortening. It was apparent later that we cannot depend only on eponyms to describe the fracture as fracture patterns and geometries include a more comprehensive range than the eponyms described. Thus, multiple classification systems were created and reported. As previously described by the international distal radius fracture study group, 15 classification systems were described in the literature. These systems were then filtered down to eight, excluding those that lacked reliability and reproducibility. These classification systems include the AO/ASIF, Fernandez, Frykman, Gartland, Mayo, Melone, Older’s, Werley, and Universal classifications systems. These systems differ; for example, Frykman focused on the involvement of the radiocarpal joint, radioulnar joint, and ulnar styloid. In contrast, the AO/ASIF is more descriptive and has multiple subdivisions that aid in choosing the treatment methodology [3-6]. Although historically, there was much emphasis on the use and development of classification systems in DRF, Shehovych et al. concluded in their study that there was inadequate evidence to demonstrate their clinical usefulness [3].

DRF treatment has evolved over the years, aiming to deliver the best possible functional outcome to the patient. The treatment is selected based on multiple factors, such as fracture location, geometry, soft tissue status, and the patient’s age, among other factors. The most common treatment method is cast application. Immobilizing the fracture minimizes the strain at the fracture site, allowing proper fracture healing and union [4,5].

The treatment spectrum ranges from non-operative to operative intervention. Many options constitute this spectrum as it was laid out by Goldfarb et al.. These include (I) closed reduction; (II) closed reduction with percutaneous pinning; (III) closed reduction with percutaneous pinning and external fixation, with or without the usage of bone graft; (IV) closed reduction and external fixation; (V) open reduction with percutaneous pinning; (VI) open reduction with plate fixation; (VII) open reduction with external fixation; (VIII) limited open reduction (lunate facet) with percutaneous pinning; (IX) limited open reduction (lunate facet) with plate fixation; and (X) limited open reduction (lunate facet) with external fixation [6,7].

Regardless of the stabilization method, the fracture will require a period of immobilization and motion limitation to allow proper fracture healing, reduce pain and minimize displacement. Immobilization is usually achieved by various methods, whether applying a cast, a splint, or a brace [4,8,9]. The period of immobilization differs in operative and non-operative patients. Although there is no consensus regarding the optimal immobilization period for non-operative treatment of DRF, the usual time frame is between 4-6 weeks of immobilization [10-15]. Patients remain immobilized for six weeks in our hospital and then start physiotherapy. However, some patients exceed the six-week immobilization period due to multiple factors. These can be attributed to either patient factors, physician factors, the fracture itself, or a combination of the previous. Patient factors include incompliance to treatment, low socioeconomic status, or loss of follow-up. Physicians sometimes delay mobility based on the chosen treatment method and the patient’s general status. In some situations, immobilization is continued if no signs of healing are shown radiologically.

Our study wanted to see the impact of prolonged immobilization on the patients' hand function if they exceeded six weeks. The study results can then be utilized and translated to patients during their management to explain the benefits and risks objectively.

A period of 4-6 weeks will be considered in our study as the “standard” period of immobilization. If immobilization exceeds this time frame, it will be regarded as “prolonged” immobilization.

Since hands play an essential role in our daily lives and are often the means to one’s livelihood, prolonged immobilization post-DRF negatively affects a patient’s life quality. To help mitigate these complications, starting physical rehabilitation (PR) and occupational therapy (OT) as early as possible is preferred. Assessment and evaluation of hand function are first done to establish a baseline. Range of motion (ROM), defined as the joint’s entire potential movement, is measured by Goniometer. Jamar dynamometer is also used for assessing hand strength [6,7].

There are no clear specific statistics on prolonged immobilization effects on hand functions post DRF in the Gulf countries. The lack of documented data is the primary reason behind the insufficiency. Therefore, a study on patients who had DRF and underwent various periods of immobilization measures the effects on hand functions according to the measurements done by the OT. The study aims to detect how prolonged immobilization impacts the patient’s hand functions post DRF at King Abdulaziz Medical City in Jeddah.

## Materials and methods

Study design

A retrospective cohort study was conducted to compare immobilization per protocol (4-6 weeks of immobilization) and deviated from protocol (more than six weeks of immobilization) patients post distal radial fracture. We reviewed the file of patients who had DRF with the X-rays and filtered those who fit our inclusion criteria. OT team collected the hand function measurements retrospectively using a functional standard form for both groups. Data was then analyzed to detect if prolonged immobilization affected patients' hand functions.

Materials and methods

The sample was collected by non-probability convenience sampling method, according to the availability and accessibility of the data. The data collected was from the medical records at King Abdulaziz Medical City - Jeddah from December 2016 to December 2019. The study involved human subjects and therefore we have acquired approval from the Institutional Review Board (IRB) except for informed consent. However, data and patient privacy were confidential and all data was then provided on an excel sheet with randomly assigned numbers to patients with no names provided on the sheet. Entering medical terms such as polytrauma, multiple fractures, and distal radius fracture was considered the basic to search the population. The population was distal radial fracture adult patients in the rehabilitation department at King Abdulaziz Medical City in Jeddah. Patients who did not meet the inclusion criteria were excluded. The included participants were divided into two groups, a group that was mobilized early and a group that had prolonged immobilization. Afterward, the information of both groups was analyzed to determine if the period of immobilization affected hand functions or not.

Inclusion and exclusion criteria

The study inclusion criteria were: (1) males and females adults post-DRF (18 years old and above), (2) patients with unilateral or bilateral DRF involvement, (3) patients with polytrauma that includes distal radial fracture, (4) patients with multiple fractures that include distal radial fracture, (5) patients with a distal radial fracture who were treated initially at King Abdulaziz Medical City, or who were referred from another hospital and follow-up data was available, and (6) patients with a distal radial fracture who were treated by an operative or non-operative treatments.

The exclusion criteria of the study were: (1) patients with pathological distal radial fracture, (2) patients with distal radial fractures with associated ulnar fracture, carpal bone injury, or distal radial, ulnar joint injuries.

Sample size

The sample was divided into two groups (Per-protocol and deviated from protocol). For the sample size calculation for the study of distal radial fracture to detect the difference between the prevalence of loss of hand function of 10% in the per-protocol group, in comparison to the loss of 40% of hand function in the deviated from protocol group, with D = (0.03), alpha = 5%, and 20% power, we needed to recruit 76 patients distributed into two groups: (1) Per-protocol = 38 patients, (2) Deviated = 38 patients.

Data collection methods used and measurement

A data collection sheet was developed to collect the patients’ data. The questions of the data collection sheets were categorized into three sections: 1) demographic questions including gender and age, 2) fracture information including date of injury, date of definitive intervention, fracture description, date of the initial assessment, type of intervention, period of immobilization, 3) hand functions measurements including goniometry measurements by degrees, Jamar dynamometer measurements by kilograms, and pain rate by numerical visual analog scale.

Data management and analysis plan

SPSS version 2017 (IBM Corp., Armonk, NY) was used for the statistical analysis of this study; it analyzed the collected data. For qualitative data, percentages and frequencies were used. Mean, median, and standard deviation were applied to summarize the simple descriptive statistics for quantitative data. For bivariate analysis, we compared the outcomes of hand function between the per-protocol group and the group that deviated from protocol. The study outcomes (chi-square) test was used with a significant level set at <0.05, and a t-test was used to compare qualitative and quantitative variables.

## Results

Out of the screened patients, 44 patients met the inclusion criteria, of which 25 (56%) were women, and 19 (44%) were men. Participants had a mean age of 47.8 years. Intra-articular DRF was diagnosed in 19 (44%) of participants, while the remainder had extra-articular (55%). Patients were treated directly with the cast with no need to attempt reduction in 17 (38%) of the cases. Reduction of a displaced fracture followed by cast application was made in 15 (34%) of the patients, and 12 (27%) underwent operative treatment. Immobilization of the fracture was per-protocol in 20 (46%) of the participants, and 24 participants (54%) deviated from protocol.

The functional outcomes for each group are presented in Table 1. The results of the ROM tests showed that the patients with immobilization that deviated from protocol had a non-significant limitation in their extension movement (P = 0.641) compared to the per-protocol group. Flexion movement was affected in both groups, and the difference was not significant (P = 0.674). Participants whose immobilization deviated from protocol had limitations in ulnar deviation movement (P = 1.000) compared to the per-protocol group. In radial deviation movement, participants in both groups had limitations (P = 1.000). Regarding forearm rotation, participants whose immobilization deviated from protocol had limitations in supination (P = 0.575) and pronation (P = 0.370) (31.9%). The per-protocol group had pain compared to deviated from the protocol group (68.1%) (P = 0.263). Similarly, participants whose immobilization deviated from protocol showed decreased grip strength (P = 0.291) (Table 2).

**Table 1:** Hand function in both groups ROM: Range of motion

Fracture	Per protocol		Deviated from protocol		P-value
	Functional ROM	Limited ROM	Functional ROM	Limited ROM	
Flexion	60.0%	58.8%	40.0%	41.2%	0.674
Extension	57.1%	58.1%	42.9%	41.9%	0.641
Supination	50.0%	55.6%	50.0%	44.4%	0.575
Pronation	46.2%	66.7%	53.8%	33.3%	0.370
Radial Deviation	50.0%	50.0%	50.0%	50.0%	1.000
Ulnar Deviation	50.0%	50.0%	50.0%	50.0%	1.000

**Table 2:** Grip and Pain outcome in both groups

		DC Cast								
		Per protocol		Deviated from protocol		Total	p-value	OR	95% CI	
		n	%	n	%					
Grip rate										
	Weak	13	68.4%	6	31.6%	19	0.291	4.333	0.326	57.649
	Normal	1	33.3%	2	66.7%	3				
Pain Rate										
	Severe	9	52.9%	8	47.1%	17	0.263	cannot be computed		
	Moderate	8	80.0%	2	20.0%	10				
	Mild	1	33.3%	2	66.7%	3				

## Discussion

DRF is a common orthopedic fracture in both the young and elderly. The usual mechanism of injury is falling on an outstretched hand. Depending on the mechanism of injury, different fracture patterns and types can occur. Treatment of distal radius varies from non-operative to operative options depending on multiple factors. Duration of immobilization post-treatment is required whether the treatment was operative or not. There is no agreed period in the literature for the immobilization period in both treatment methods [5,6].

Non-operative treatment includes either a direct cast application or a more invasive method where the fracture undergoes a trial of closed reduction and casting. Most fractures show great functional results when treated non-operatively and casted with Plaster of Paris [7,8]. The cast is then molded using a three-point fixation method to prevent displacement and counter the deforming forces holding the reduction in place [6]. The hand is then placed in a slightly flexed and ulnar-deviated position. This method and position were made popular by Charnley in 1950 and have been used since then [9]. Furthermore, if the fracture is reduced in a closed manner, then casted, post-reduction X-rays should be reviewed to determine whether the reduction and cast positioning are acceptable and adequate.

The patients will be closely followed up to ensure that the fracture remains non-displaced and heals within the acceptable parameters. The highest risk of fracture displacement is within the first two weeks. Only 7% to 8% of DRF displace after the first two weeks [10]. The period of immobilization and time for cast removal has been discussed extensively in the literature. The common practice prefers a 4-6 weeks immobilization period, although sufficient and convincing evidence is lacking to support this duration [11].

Bentohami et al. conducted a prospective randomized controlled trial in which they compared three and five weeks cast immobilization for treatment of a non or minimally displaced DRF in the adult population. The study showed that the three weeks immobilization group had a significantly better Patient-Related Wrist Evaluation and Quick Disability of Arm, Shoulder, and Hand scores after one-year follow-up. Moreover, both periods showed similar results in displacement and pain. Therefore, they recommended discontinuation of the cast after three weeks [12,13]. A recent systemic review with level II evidence conducted by Van Delft et al. compared multiple prospective studies and randomized controlled trials. Their systemic review compared a non-operative group that underwent three weeks or less of immobilization to another non-operative group with a regular period of immobilization. It was shown that the former group showed a functional outcome that was non-inferior to the group with a regular period of immobilization. The result suggests that three weeks of immobilization might result in better functional outcomes due to a shorter period of immobilization. Also, they have recommended the need for future studies to have a clear conclusion of the optimal period of immobilization [12-15].

Operative options and interventions vary, as mentioned previously, with each intervention having its indication, advantages, and disadvantages. As noted by The American Academy of Orthopedic Surgeons (AAOS) in 2009, operative interventions are indicated in distal radius when dorsal tilt is more than 10°, intra-articular displacement or step-off is more than 2 mm, radial shortening is more than 3 mm. Furthermore, irreducible or unstable fractures, open fractures, DRF associated with carpal fracture, fractures in polytrauma patients, and fractures that failed conservative treatment indicate surgery [16-17]. Some studies have focused on the surgical treatment option in the elderly population. A systematic review and meta-analysis compared operative and non-operative treatment in patients 60 years old or more. Their results showed that although operative groups showed better wrist function, it was not statistically significant, and both groups had similar ROM at the final follow-up. Radiographic outcomes were statistically significant in the operative group, but the relationship between clinical effect and image is still not established. Thus, the authors concluded no difference in functional outcome between both groups [18].

For the operative group, immobilization is required as well post-operation. We reviewed three randomized control trials comparing different periods of immobilization to see the effect on hand function and determine the optimal period of immobilization post-fixation [19-22].

Quadlbauer et al. conducted a randomized controlled trial (RCT) to compare functional results between immediate mobilization post-op for distal radius fracture and five weeks immobilization. Their study found that the rapid mobilization group has a statistically significantly better ROM in the sagittal plane and grip strength in the immediate mobilization group during the first six months after the surgery. Extension and forearm rotation were also significantly better results in the first six weeks, while flexion in the first nine weeks after the surgery. In the frontal plane, significantly better results were found in the immediate mobilization group during the first nine weeks after the surgery. The study is likely underpowered with its small sample and lack of sample size calculation. They mentioned that further studies on a larger population should be conducted [19-20].

Among the other randomized control trials, Watson et al. conducted the most extensive randomized control study on groups who sustained a distal radius fracture and were treated operatively. Their study aimed to determine the optimal period for immobilization post-fracture fixation. They compared hand function, range of motion, and pain primarily among immobilization periods of one, three, and six weeks post-fixation. All groups underwent physiotherapy post-splint removal. They found no significant statistical difference between these three groups regarding hand function, range of motion, and pain after three and six months post-operation. Although statistical differences were noted in the one and three weeks group compared to the six weeks group, they concluded that no differences were noted in terms of long-term outcomes [21].

Lozano et al. hypothesized that after distal radius fracture fixation, mobilization of the joint after two weeks or six weeks would not significantly affect the flexion-extension arc at three and six months after surgery. A randomized controlled study was done for 60 patients. No significant difference was found between the two groups at three and six months after surgery regarding hand function, ROM, pain, and radiological parameters. Their data concluded that six weeks of immobilization does not compromise hand function and DASH scores compared to two weeks [22].

There are many unique qualities in the study: (1) It is one of the first studies conducted about this topic in the Gulf countries; (2) it tackles one of the most common cases treated in the OT department; (3) the study excludes patients who have characteristics that might affect the measurements, such as associated ulnar shaft fracture, carpal bone fractures, distal radioulnar joint injuries, and pediatric patients; (4) standardized and non-standardized measures were used to assess hand functions.

Confining our study in King Abdulaziz Medical City in Jeddah limited us from achieving the sample size required. Another limitation is some missing data necessary for the data collection sheet that OT did not document.

P-values are highly influenced by sample size; thus, the larger the sample size, the more likely the P-value will be significant and vice versa. Interpretations of clinical studies should not depend only on P-value results as results may appear significant in terms of P-value but have a minimal clinical effect [23]. Although the full sample size was not collected, the study provided evidence to support the importance of following the immobilization protocol for better hand function outcomes. The results showed a correlation between the period of immobilization and hand function post-DRF.

## Conclusions

DRFs are among the most common orthopedic cases in the emergency room and one of the most common referred cases to the OT department. Cast treatment was more common among the participants. After treatment, 20 (46%) of the participants were immobilized as per protocol (six weeks), and 24 (54%) of them deviated from the protocol (>6 weeks). Patients with prolonged immobilization had statistically insignificant limited ROM in supination and pronation. However, the protocol group had better ROM in all movements. A decreased grip strength and higher pain rate were observed in those patients with prolonged immobilization, compared to the per-protocol group. Therefore, an association was found between the period of immobilization and hand functions; people with more than six weeks of immobilization had limited hand functions.

## References

[1] Nellans KW, Kowalski E, Chung KC (2012). The epidemiology of distal radius fractures. Hand Clin.

[2] O'Neill TW, Cooper C, Finn JD (2001). Incidence of distal forearm fracture in British men and women. Osteoporos Int.

[3] Shehovych A, Salar O, Meyer C, Ford DJ (2016). Adult distal radius fractures classification systems: essential clinical knowledge or abstract memory testing?. Ann R Coll Surg Engl.

[4] Raittio L, Launonen A, Hevonkorpi T (2017). Comparison of volar-flexion, ulnar-deviation and functional position cast immobilization in the non-operative treatment of distal radius fracture in elderly patients: a pragmatic randomized controlled trial study protocol. BMC Musculoskelet Disord.

[5] Schneppendahl J, Windolf J, Kaufmann RA (2012). Distal radius fractures: current concepts. J Hand Surg Am.

[6] Goldfarb CA, Yin Y, Gilula LA, Fisher AJ, Boyer MI (2001). Wrist fractures: what the clinician wants to know. Radiology.

[7] McQueen M, Caspers J (1988). Colles fracture: does the anatomical result affect the final function?. J Bone Joint Surg Br.

[8] Cooney WP (1989). Management of Colles’ fractures. J Hand Surg.

[9] Charnley J (2005). The Closed Treatment of Common Fractures.

[10] Solgaard S (1986). Early displacement of distal radius fracture. Acta Orthop Scand.

[11] Christensen OM, Christiansen TG, Krasheninnikoff M, Hansen FF (1995). Length of immobilisation after fractures of the distal radius. Int Orthop.

[12] Bentohami A, de Korte N, Sosef N, Goslings JC, Bijlsma T, Schep N (2014). Study protocol: non-displaced distal radial fractures in adult patients: three weeks vs. five weeks of cast immobilization: a randomized trial. BMC Musculoskelet Disord.

[13] Bentohami A, van Delft EA, Vermeulen J (2019). Non- or minimally displaced distal radial fractures in adult patients: three weeks versus five weeks of cast immobilization-a randomized controlled trial. J Wrist Surg.

[14] Christersson A, Larsson S, Sandén B (2018). Clinical outcome after plaster cast fixation for 10 days versus 1 month in reduced distal radius fractures: a prospective randomized study. Scand J Surg.

[15] van Delft EA, van Gelder TG, de Vries R, Vermeulen J, Bloemers FW (2019). Duration of cast immobilization in distal radial fractures: a systematic review. J Wrist Surg.

[16] Lichtman DM, Bindra RR, Boyer MI (2010). Treatment of distal radius fractures. JAAOS - J Am Acad Orthop Surgeons.

[17] Alluri RK, Hill JR, Ghiassi A (2016). Distal radius fractures: approaches, indications, and techniques. J Hand Surg Am.

[18] Chen Y, Chen X, Li Z, Yan H, Zhou F, Gao W (2016). Safety and efficacy of operative versus nonsurgical management of distal radius fractures in elderly patients: a systematic review and meta-analysis. J Hand Surg Am.

[19] MacIntyre NJ, Dewan N (2016). Epidemiology of distal radius fractures and factors predicting risk and prognosis. J Hand Ther.

[20] Quadlbauer S, Pezzei C, Jurkowitsch J (2017). Early rehabilitation of distal radius fractures stabilized by volar locking plate: a prospective randomized pilot study. J Wrist Surg.

[21] Watson N, Haines T, Tran P, Keating JL (2018). A comparison of the effect of one, three, or six weeks of immobilization on function and pain after open reduction and internal fixation of distal radial fractures in adults: a randomized controlled trial. J Bone Joint Surg Am.

[22] Lozano-Calderón SA, Souer S, Mudgal C, Jupiter JB, Ring D (2008). Wrist mobilization following volar plate fixation of fractures of the distal part of the radius. J Bone Joint Surg Am.

[23] Marks M, Rodrigues JN (2017). Correct reporting and interpretation of clinical data. J Hand Surg Eur Vol.

