# Correction: Comparison of the Effects of Regular Periods of Immobilization and Prolonged Immobilization on Hand Function Post Distal Radial Fracture

**DOI:** 10.7759/cureus.c134

**Published:** 2023-08-31

**Authors:** Mohammed Khashab, Ahmed Alem, Alhanouf Almuatiri, Fatmah Rasheed, Mai Almehmadi, Shahad Felemabn, Samah Gassass, Majed Alosaimi, Hani Sulimani, Ali Alyami

**Affiliations:** 1 Orthopedics, King Abdulaziz Medical City, Jeddah, SAU; 2 Research Office, King Abdullah International Medical Research Center, Jeddah, SAU; 3 Orthopedics, College of Medicine, King Saud bin Abdulaziz University for Health Sciences, Jeddah, SAU; 4 Orthopaedic Surgery, King Saud Bin Abdulaziz University for Health Sciences College of Medicine, Jeddah, SAU; 5 Occupational Therapy, King Saud Bin Abdulaziz University, Jeddah, SAU; 6 Health Sciences, King Saud Bin Abdulaziz University, Jeddah, SAU; 7 Orthopaedic Surgery, King Abdulaziz Medical City/Ministry of National Guard - Health Affairs, Jeddah, SAU; 8 Orthopaedic Surgery, King Abdullah International Medical Research Center, Riyadh, SAU; 9 Orthopaedic Surgery, King Saud Bin Abdulaziz University for Health Sciences, Jeddah, SAU; 10 Orthopaedics, King Saud Bin Abdulaziz University for Health Sciences College of Medicine, Jeddah, SAU; 11 Orthopaedics, King Abdulaziz Medical City/Ministry of National Guard - Health Affairs, Jeddah, SAU; 12 Surgery/Musculoskeletal Oncology, Limb Reconstructive Surgery, Sport Medicine and Arthroscopy, King Saud Bin Abdulaziz University for Health Sciences, Jeddah, SAU

This article has been corrected at the request of the authors to correct the affiliation for Mohammed Khashab from Orthopaedic Surgery, King Abdulaziz Medical City/Ministry of National Guard - Health Affairs, Jeddah, SAU to:

1. Orthopedics, King Abdulaziz Medical City, Jeddah, Saudi Arabia

2. Research Office, King Abdullah International Medical Research Center, Jeddah, Saudi Arabia.

3. Orthopedics, College of Medicine, King Saud bin Abdulaziz University for Health Sciences, Jeddah, Saudi Arabia

The authors deeply regret that these errors were not identified and addressed prior to publication.

 

